# The effect of sulindac, a non-steroidal anti-inflammatory drug, attenuates inflammation and fibrosis in a mouse model of chronic pancreatitis

**DOI:** 10.1186/1471-230X-12-115

**Published:** 2012-08-24

**Authors:** Han Bai, Xiaokai Chen, Lin Zhang, Xiaoguang Dou

**Affiliations:** 1 Department of Infectious Disease, Shengjing Hospital of China Medical University, Shenyang, 110004, China; 2 Department of Surgery, Shengjing Hospital of China Medical University, Shenyang, 110004, China

**Keywords:** Sulindac, Chronic pancreatitis, Chemoprevention

## Abstract

**Background:**

Chronic pancreatitis is characterized by progressive fibrosis, pain and loss of exocrine and endocrine functions. The long-standing chronic pancreatitis and its associated pancreatic fibrosis are the most common pathogenic events involved in human pancreatic carcinogenesis, but the therapeutic strategies to chronic pancreatitis and the chemoprevention of pancreatic carcinogenesis are very limited.

**Methods:**

We investigated the effect of sulindac, a non-steroidal anti-inflammatory drug (NSAID), on inhibition of chronic pancreatitis in a caerulein induced chronic pancreatitis mouse model.

**Results:**

Sulindac significantly reduced the severity of chronic pancreatitis including the extent of acini loss, inflammatory cell infiltration and stromal fibrosis. The protein expression of phosphorylation of MEK/ERK was inhibited in the chronic pancreatic tissues by sulindac treatment as measured by Western blot assay. The levels of inflammatory cytokines including TNF-α and MCP-1 were also significantly decreased with sulindac treatment, as well as the expression of TGF-β, PDGF-β, SHH and Gli in the chronic pancreatic tissue detected by qPCR assay and confirmed by western blot assay. The activation of pancreatic satellet cells was also inhibited by sulindac as measured by the activity of α-smooth muscle actin (α-SMA) in the pancreatic tissue of chronic pancreatitis.

**Conclusions:**

Sulindac is a promising reagent for the treatment of chronic pancreatitis via inhibition of inflammatory cell infiltration and stromal fibrosis, the inhibitory effect of sulindac on chronic pancreatitis may through targeting the activation ERK/MAPK signaling pathway.

## Background

Chronic pancreatitis (CP) is a disease with a succession of pathophysiological events including inflammatory cell infiltration, parenchymal atrophy, and extensive fibrosis of the exocrine pancreas [1]. The pathophysiology of chronic pancreatitis is not completely understood, although a number of advances have been made in recent years. The most accepted theory is that repeated acute attacks of pancreatic necro-inflammation together with a dysregulated ability to repair organ damage leads to activation of a fibrotic cascade and loss of parenchymal mass [2].

Pancreatic stellate cells (PSCs) play a crucial role in the initiation and progression of pancreatic fibrogenesis in chronic pancreatitis [3]. When PSCs are stimulated by the profibrogenic mediators, the quiescent cells converts to a myofibroblast-like, alpha smooth muscle actin (a-SMA)-positive cells that is highly proliferative and capable of depositing fibrillar collagen in the interstitial spaces [4]. PDGF and TGF have been shown to have a significant impact on the activity of PSCs that are generated by inflammatory cells [5]. PDGF appears to be a potent mitogen and chemoattractant for PSC, whereas TGF-β stimulates PSCs to synthesize and secrete matrix proteins such as type 1 collagen, fibronectin and laminin, and MMP 2, 3 and 13 [6-8].

Hedgehog signal is frequently activated in fibrogenic pancreatic diseases such as chronic pancreatitis [9], the molecules of Hedgehog pathway are frequently up-regulated in the conditions of these diseases, which indicated that hedgehog signal is involved in pancreatic fibrogenesis, and enhanced migration of pancreatic stellate cells maybe one of the mechanisms [10].

Despite the better understanding of its mechanisms, the therapeutic strategies to CP are mostly symptomatic and very limited. Cycloxygenase (COX) is a key enzyme in the synthesis of prostaglandins (PGs) from arachidonic acid [11]. Elevated COX-2 levels have been identified both in pancreatic tissue from patients with chronic pancreatitis and animal model of chronic pancreatitis [12-14]. PGE2 is a known proinflammatory molecule that has been studied in many chronic diseases, including pain, osteoarthritis, and rheumatoid arthritis [15-17], as well as in cancer [18,19], and hence providing a potential target for the treatment of CP. COX-2 inhibitors, inhibit the activities of cyclooxygenases (COXs), significantly suppresse inflammation and fibrosis and delay the progression of the diseases [1,14,20,21]. Sulindac is a non-steroidal anti-inflammatory drug (NSAID) of the arylalkanoic acid class that, in vivo, is reversibly converted into its anti-inflammatory active compound, sulindac sulfide, with the biological effects of inhibition on both cyclooxygenase-1 (COX-1) and COX-2 activities and reduction in prostaglandin (PG) synthesis [22].

There are several animal models for chronic pancreatitis, repeatedly caerulein intraperitoneal injection induced mice chronic pancreatitis is one of the best characterized chronic pancreatitis mouse model [23,24]. Caerulein is a cholecystokinin analog that is commonly used to induce either acute or chronic pancreatitis. Cholecystokinin is a major physiologic regulator of digestive enzyme secretion by the pancreatic acinar cell , however, supramaximal stimulation by cholecystokinin and its analogs could generate a distinct pancreatic response that includes diminished secretion, accumulation of secretory proteins within the pancreas and cause pancreatic injury [25], Chronic fibrotic changes in the pancreas are to perform self-limited acute acinar cell injury by repeated pathological stimuli of the gland by caerulein [26]. This method mimics the clinical observation that repeated episodes of acute pancreatitis, irrespective of its origin, lead to increasing damage of the organ that eventually results in atrophy and fibrosis [3]. The induction of acute or chronic pancreatitis by caerulein depends on the schedule and the dose administrated [24,27].

In the present study, we examined the therapeutic effect of sulindac on chronic pancreatitis using caerulin-induced chronic pancreatitis mouse model, the effect of sulindac on the development of chronic pancreatitis was examined. Systemically histopathologic and immunohistochemical analysis of chronic pancreatitis, and chronic inflammation activated signals were also performed.

## Materials and methods

### Animal experiment

Thirty C57BL/6 mice of 6–8 weeks old were obtained from Shanghai Experimental Center of Chinese Academy of Science (Shanghai, China). Mice were housed under pathogen-free conditions with free access to water and lab chow in the facilities of Laboratory Animal Services, Shengjing Hospital of China Medical University. After 1 week of new arrival, the mice were enrolled in the experiment with both genders. All studies were conducted in compliance with the China Medical University Institutional Animal Care and Use Committee (IACUC) guidelines.

Sulindac (purity ≥ 98%) and caerulein (purity ≥ 97% by HPLC) were purchased from Sigma Aldrich (Shanghai, China). Based on literatures, Chronic pancreatitis was induced by repeated intraperitoneal (IP) injections of caerulein with 50 μg/kg every hour for 6 hours, twice weekly, for 10 weeks in 20 mice, 10 mice treated with saline IP injected instead of caerulein were as control [26]. AIN-76A diet (control diet) and AIN-76A diet supplemented with 200 ppm sulindac (sulindac diet) were from Trophic animal feed high-tech Co., Ltd. (Nantong, China). All mice received AIN-76A diet for the first 2 weeks after caerulein injection, and then the mice were administrated with either sulindac diet (10 caerulein treated mice and 5 control mice) or control diet (10 caerulein treated mice and 5 control mice) for 8 weeks with free access to water. The dose of sulindac used in this study was based on the literature [28] and the previous experience of study on sulindac in our lab. The diets were replenished every 3 days, food and water consumption were monitored every day and body weight was measured weekly.

### Tissue Preparation and histological analysis of chronic pancreatitis

At the end of the experiment, the mice were sacrificed by CO_2_ asphyxiation; pancreases and other key organs such as liver and spleen were collected and weighted. Half of the pancreas was fixed in 10% buffered formalin for 24 h, routinely processed, and embedded in paraffin, the other part were stored freshly at −80°C for protein and RNA assay. Serial paraffin sections (5 μm) were made and stained with hematoxylin and eosin (H&E) for histopathological examination. Additional tissue sections were obtained on poly-L-lysine coated slides for histochemical staining (Masson Trichrome) and immunohistochemical staining.

Chronic pancreatitis was blindly analyzed and graded using a semi-quantitative scoring system according to our published paper [29]. The criteria for chronic pancreatitis scoring were summarized in table 1. The chronic pancreatitis index (CPI) was expressed as a sum of scores on loss of acini, inflammatory cell infiltration and stromal fibrosis. The extent or areas of loss of acini were evaluated of the whole pancreas area; the number of inflammatory cells infiltration was measured for at least 10 non-overlapping and randomly selected fields per high power field (40X objective lens) and the area and intensity of stromal fibrosis of the pancreas was graded based on Masson Trichrome staining.

**Table 1:** Scoring criteria for grade of chronic pancreatitis

	**Extent or areas of loss of acini**	**Inflammatory cell infiltration**	**Intensity of stromal fibrosis**
0	absent	<5/High power field	absent
1	<10%	5-50/High power field	<5%
2	10-30%	50-100/High power field	5-15%
3	>30%	>100/High power field	>15%

### Immunohistochemical analysis

Immunohistochemical staining was performed according to the routine protocol using an avidin-biotin-peroxidase method [30]. Briefly, Sections were deparaffinized and rehydrated, the sections were then boiled with antigen unmasking solution to retrieve the antigens, and were quenched with 3% hydrogen peroxide. After blocked with normal serum, the sections were incubated with one of the primary antibodies including rabbit polyclonal anti-myeloperoxidase (MPO) antibody (dilution 1:35; Abcam, Cambridge, Massachusetts, USA.), rat anti-murine Mac-3 monoclonal antibody (dilution 1:20; Novus Biologicals, CO, USA), rabbit monoclonal anti-phospho-ERK1/2 (dilution 1:100; Cell Signaling Technology, Boston, Massachusetts, USA) antibody at 4°C over night. Then after incubation with the appropriate biotinylated secondary antibody (dilution 1:200) and avidin-biotin-peroxidase complex, a characteristic brown color was developed by incubation with 3, 3-diaminobenzidine substrate chromogen system (Sigma-Aldrich, Shanghai, China).

A sequential method for Amylase/CK-19 double staining was used according to the immunohistochemistry enzyme double staining protocol [31]. Same as described above, the sections were incubated with goat polyclonal anti-CK19 antibody (dilution 1:50; Santa Cruz Biotechnology, Santa Cruz, California, USA) as the first primary antibody and detected by DAB substrate chromogen system (Sigma-Aldrich). the sections were then blocked again with normal serum, and incubated with the second primary antibody, mouse monoclonal anti-Amylase antibody (dilution 1:50; Santa Cruz Biotechnology, Santa Cruz, California, USA), after incubating with the anti-mouse secondary antibody and avidin-biotin-peroxidase complex, 3-amino-9-ethylcarbazole (AEC) peroxidase substrate with a characteristic red color was used to detect the positive staining and distinguish from the brown color of DAB.

The negative control was established by replacement of primary antibody with normal serum, and appropriate positive control was used for each primary antibody. Specific antibody-labeled signals were analyzed under Nikon research microscope.

### Quantitative real-time PCR Assay

Total RNA was extracted from fresh pancreatic tissue using RNeasy® mini kit (Qiagen, Inc., Valencia, CA, USA). RNA concentration was determined using a SmartSpec Plus Spectrophotometer (BioRad, Hercules, CA, USA). First-strand cDNA was synthesized using 1 μg of total RNA in a 20 μl reverse transcriptase reaction mixture using iScriptTM cDNA synthesis kit (BioRad) according to the manufacturer’s instructions. The regions of Tumor necrosis factor α(TNF- α), Monocyte chemotactic protein 1 (MCP-1), Transforming growth factor β (TGF-β), Platelet derived growth factor β (PDGF-β), Sonic hedgehog homolog (Shh) and Glioma-associated oncogene homolog 1 (GLI1), and also alpha-smooth muscle actin (α-SMA) mRNA were amplified using the following primers shown in table 2 which were designed using the primer 3 software. Glyceraldehyde-3-phosphate dehydrogenase (GAPDH) was used as internal control. All Real-Time PCR reactions were performed in a 20 μl mixture containing 1/10 volume of cDNA preparation (2 μl), 10 μl iQTM SYBR® Green supermix (BioRad), 0.5 μM of each primer and 8 μl diethylprocarbonate (DEPC) water. Real-time quantitation was performed using the MiniOpticon Real-Time PCR System (BioRad). PCR conditions were: 50°C for 2 minutes, 95°C for 2 minutes, followed by 40 cycles of 95°C, 15 sec; 58°C, 3 sec; 50°C, 1 sec. Data of each mRNA expression were shown as the relative folds of change normalized by that of GAPDH.

**Table 2:** Primer Sequences of Genes Evaluated by Real-time Quantitative PCR

**Gene**	**Forward sequence**	**Reverse sequence**
TNF-α	5’-CCAACGGCATGGATCTCAAAGACA	5’-TGAGATAGCAAATCGGCTGACGGT
MCP-1	5’-ACTGAAGCCAGCTCTCTCTTCCTC	5’-TTCCTTCTTGGGGTCAGCACAGAC
TGF-β	5’- TTGCTTCAGCTCCACAGAGA	5'-TGGTTGTAGAGGGCAAGGAC
PDGF-β	5’-CCCACAGTGGCTTTTCATTT	5'-GTGGAGGAGCAGACTGAAGG
SHH	5’-CCTCTCCTGCTATGCTCCTG	5’-GTGGCGGTTACAAAGCAAAT
GLI1	5’-ACTAGGGGGCTACAGGAGGA	5’-ACCTGGACCCCTAGCTTCAT
α-SMA	5’-GGCTCTGGGCTCTGTAAGG	5’-CTCTTGCTCTGGGCTTCATC
GAPDH	5’-GCACAGTCAAGGCCGAGAAT	5’-GCCTTCTCCATGGTGGTGAA

### Protein extraction and Western blot assay

Freshly harvested pancreases were homogenized and lysed in ice-cold RIPA lysis buffer (Santa Cruz) for 60 minutes by vortexing every 5 min. The lysates were separated by centrifugation at 14,000 × g for 10 minutes at 4°C. The supernatants were collected, aliquot, and stored at −80°C until Western blot assay conducted. All protein concentrations were determined using the Bradford reagent.

30 μg of the total protein per lane was separated by 10% or 12% SDS-PAGE gel based on the molecular weight of the target proteins , and were then transferred onto a polyvinylidene fluoride (PVDF) membrane, after blocking with 5% bovine serum albumin (BSA) in 1X Tris-Buffered Saline with 0.1% Tween-20 (TBST) or 5% non-fat milk in TBST, the membranes were incubated with one of the following primary antibodies, including anti-phosphorate-MEK/anti-MEK, anti-phosphorate-ERK/anti-ERK, anti-TNF-α, anti-TGF- β, anti-PDGF- β, anti-MCP-1, anti-Shh and anti-GLI1 antibodies (all of the above with dilution 1:1000, Cell Signaling), and anti-α-SMA antibody (dilution 1:200; Abcam, Cambridge, Massachusetts, USA) at 4°C overnight. The membranes were then incubated with horseradish peroxidase (HRP)-linked anti-rabbit IgG or anti-mouse IgG, according to the origin of the primary antibodies, (dilution 1:2000, Cell Signaling) for 1 hour at room temperature (RT). Between the steps, the membrane was washed with TBST for 5 minutes by 3 times. The protein-antibody complex was detected by using the chemiluminescent substrate (Cell Signaling) according to the manufacturer's instructions; the emitted light was captured on an X-ray film.

### Statistical analysis

Each analyzed parameter was expressed as Mean ± SD, unless otherwise stated. Continuous variables were compared with the Student’s *t*-test, whereas categorical variables were compared with Chi-square test. All statistical tests were two-sided, statistical significance was taken as *p < 0.05*.

## Results

### Sulindac attenuated caerulein induced chronic pancreatitis in mice

**Figure 1:**
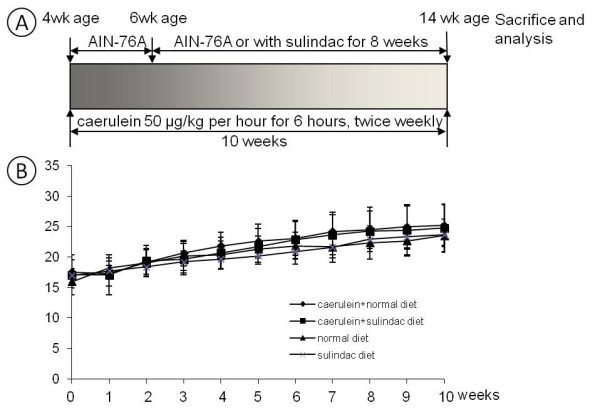
**Experiment design and monitoring of body weight during the course of the experiment.** (**A**) Experiment design for the chemoprevention of sulindac on caerulein induced chronin pancreatitis in mice. (**B**) Monitoring of body weight during the course of the experiment.

Sulindac was administered in diet for 8 weeks in this study. The average daily food consumption per mice in our experiment is about 2.6 g, the average body weight of the mice was 25.5 g, and therefore the administrated dose of sulindac per mice was about 0.5 ± 0.1 mg/day or 20 mg/kg/day. All of the Animals showed steady body weight gain during the course of the experiment. There was no significant difference of body weight in mice between caerulein induced chronic pancreatitis group and the control group, or in the mice with or without sulindac treatment. None of the animals with sulindac administration exhibited any observable toxicity or any gross changes attributable to the change in liver, spleen, kidney or lung. There were also no differences of water and food consumption among the treatment and control group (Figure 1).

**Figure 2:**
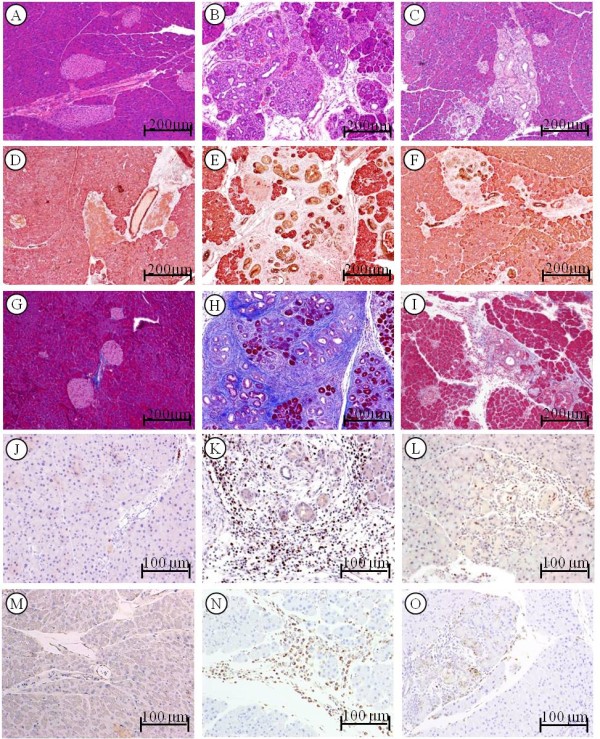
**Analysis of chronic pancreatitis using the approaches of histopathological, immunohistochemical and histochemical assay.*Histopathology in the pancreatic tissue of mice:*** (**A**) Pancreas in control mice showing morphologically normal pancreatic parenchyma (acini and islets as well interlobular ducts). (**B**) Extensive chronic pancreatitis in *caerulein induced chronic pancreatitis* mice showing loss of acini and stromal fibrosis. (**C**) Mild chronic pancreatitis in mice with sulindac treatment. ***Amylase and CK19 expression in the pancreas***: (**D**) Pancreas from control mice showing mainly composed of acinar cells and scattered islets, CK19 only positive in the ducts. (**E**) In *caerulein induced chronic pancreatitis* mice, the amylase positive acinar cells were instead by CK19 positive ductal cells. (**F**) *Chronic pancreatitis* mice treated with sulindac exhibiting increased amylase positive area and decreased CK19 positive area. ***Trichrome stain highlighting stromal fibrosis in the pancreas:*** (**G**) Trichrome stained fibroconnective tissue (blue color) only in the interlobular areas of a normal pancreas in control mice. (**H**) *Caerulein induced chronic pancreatitis* mice showing extensive fibrosis in pancreatic parenchyma. (**I**) *Chronic pancreatitis* mice with sulindac treatment exhibit much less stromal fibrosis in the pancreas. MPO***-labeled neutrophils in the pancreas:*** (**J**) Pancreas from control mice showed rare MPO-labeled neutrophils. (**K**) *Caerulein induced chronic pancreatitis* mice displayed intense MPO-labeled neutrophils in the areas of pancreatitis. (**L**) *Chronic pancreatitis* mice with sulindac treatment exhibited decreased MPO-positive neutrophils. ***Mac-3-labeled macrophages in the pancreas:*** (**M**) Pancreas from control mice showed no Mac-3-labeled inflammatory cells. (**N**) *Caerulein induced chronic pancreatitis* mice displayed intense Mac-3-labeled macrophage in the areas of pancreatitis. (**O**) *Chronic pancreatitis* mice with sulindac treatment exhibited decreased Mac-3-positive macrophages.

Hematoxylin-eosin stained slides revealed no pancreatitis in control mice either with or without sulindac treatment (Figure 2A). Repeatedly caerulein treated mice exhibited extensive chronic pancreatitis as seen in Figure 2B, including multi-focal patterns of acinar loss, stromal fibrosis, and inflammatory cell infiltration. Marked decrease improvement of chronic pancreatitis was observed in mice treated with sulindac (Figure 2C).

**Figure 3:**
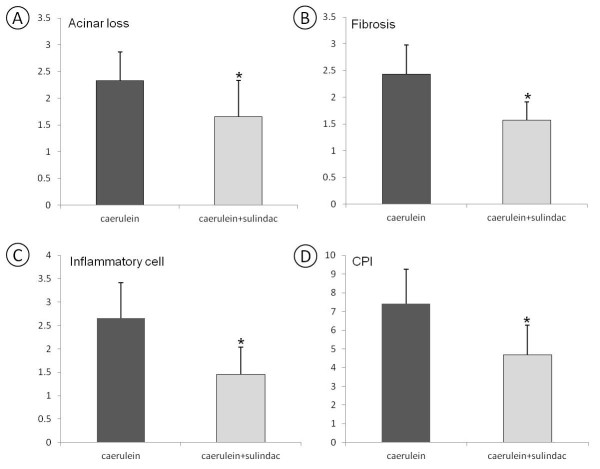
**Semi-quantitative analysis (histogram) of severity of chronic pancreatitis.** (**A**) Amylase and CK19 positive area in the pancreas showing a statistically significant difference in *caerulein induced chronic pancreatitis mice* with or without sulindac treatment (P < 0.05). (**B**) The extent of trichrome stain-labeled stromal fibrosis revealing sulindac treatment significantly decreased the extent of fibrosis in *caerulein induced chronic pancreatitis mice* (*p < 0.05*). (**C**) MPO and Mac3 labeled inflammatory cells showing a significantly decrease of neutrophils and macrophages infiltration in the pancreas of *caerulein induced chronic pancreatitis* mice with sulindac treatment *(P < 0.05)*. (**D**) Chronic pancreatitis index (CPI) as a summary of the extent of acinar loss, stromal fibrosis and inflammatory cell infiltration showed significant decrease in *caerulein induced chronic pancreatitis* mice treated with sulindac, comparing with the control mice (*p < 0.05.*)

Acinar loss and ductular proliferation were further analyzed using amylase and cytokeratin 19 (CK19) double immunohistochemical approache. In normal pancreas, amylase-labeled acinar cells were composed of about 80% of pancreatic parenchyma, and only about 2% was CK19 positive pancreatic ductal tissues (Figure 2D). A marked loss of amylase positive acinar tissues and an increase of CK19 positive ductal tissues were displayed in caerulein induced chronic pancreatitis mice (Figure 2E). Sulindac treatment significantly increased the amylase positive area of acini tissues and decreased the CK19 positive area of ductal tissues in the pancreas (Figure 2F). Semi-quantitative analysis of the amylase or CK19 positive area revealed that the average of the extent or loss of acina score was 2.33 ± 0.54 in caerulein induced chronic pancreatitis, it decreased to 1.66 ± 0.67 with sulindac treatment (Figure 3A, *P < 0.05*).

Masson Trichrome histochemical stain highlighted stromal fibrosis in chronic pancreatitis. The pancreas in control mice only showed positive trichrome’s staining in the interlobular fibro-connective tissue (Figure 2G). Extensive Trichrome positive staining was observed in the pancreatic parenchyma in caerulein treated mice (Figure 2H); decreased stromal fibrosis was observed in caerulein treated mice administrated with sulindac diet (Figure 2I). The stromal fibrosis highlighted by Masson trichrome staining was further semi-quantitatively analyzed and revealed that sulindac treatment significantly lowered the fibrosis score in the pancreas comparing with caerulein induced chronic pancreatitits mice (1.57 ± 0.34 versus 2.43 ± 0.55, *p < 0.05*, Figure 3B).

Myeloperoxidase (MPO) and Mac-3 immunohistochemical staining was used to label neutrophiles and macrophages, respectively. The pancreases in control mice exhibited no or rare MPO or Mac-3 positive cells (Figure 2J and 2M). Numerous MPO-positive neutrophiles and Mac-3 positive macrophages were observed in the pancreas of caerulein induced chronic pancreatitis mice (Figure 2K and 2N); while markedly decreased MPO and Mac-3 positive cells were observed in sulindac treated chronic pancreatitis mice (Figure 2L and 2O). MPO and Mac-3 positive cells were further semi-quantified by counting the number of positive cells per high power field (40× objective lens), as shown in Figure 3C, compared with caerulein induced chronic pancreatitis mice, a significant reduction of MPO and Mac-3 positive cells were observed in sulindac treated chronic pancreatitis mice (MPO labeled neutrophiles: 28.6 ± 18.8 versus 86.9 ± 28.5/HPF; and Mac-3-labled macrophages: 25.3 ± 16.7 versus 72.1 ± 21.2, The average inflammatory cell infiltration score was 1.46 ± 0.58 versus 2.66 ± 0.75, respectively. *p < 0.05*).

The severity of chronic pancreatitis was further semi-quantitatively analyzed based on the criteria for CPI with sum of extent or areas of loss of acini, inflammatory cell infiltration and intensity of stromal fibrosis. As shown in Figure 3D, compared to caerulin induced chronic pancreatitis mice, sulindac treated mice displayed a significant decrease of overall CPI score (4.69 ± 1.80 versus 7.42 ± 1.63, *p < 0.01*).

### Down-regulation of inflammatory cytokines and chemokines in chronic pancreatitis mice treated with sulindac

**Figure 4:**
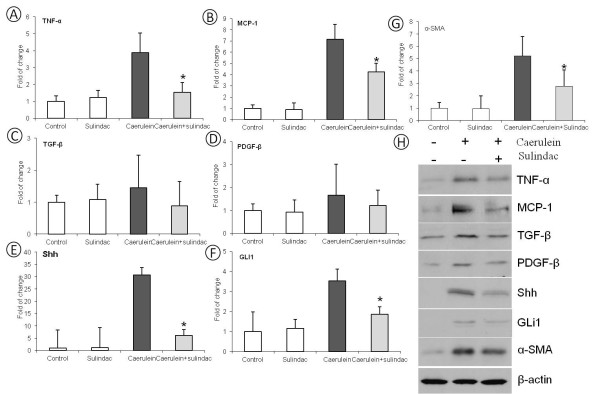
**Quantitative Real-time PCR and western blot analysis of TNF- α, MCP-1, TGF-β, PDGF-β, Shh, GLI1 and α-SMA mRNA and protein expression in the pancreas of control mice and*caerulein induced chronic pancreatitis*mice with or without sulindac treatment.** (**A**) TNF-α expression; (**B**) MCP-1 expression, (**C**) TGF-β expression, (**D**) PDGF-β expression, (**E**) SHH expression, (**F**) GLI1 expression and (**G**) α-SMA expression (*: Statistic difference, p < 0.05). (**H**) Western blot results of TNF-α, MCP-1, TGF-β, PDGF-β, SHH, GLI1 and α-SMA expression, using β-actin as loading control.

Inflammatory cytokines and chemokines were further analyzed for inflammatory activities of pancreatitis using quantitative real-time PCR approach (n = 5/group with both genders). As shown in Figure 4A-4B, the mRNA levels of TNF-α and MCP-1 in the pancreatic tissue was significantly increased in caerulein induced chronic pancreatitis mice compared to the control mice. Sulindac treatment significantly lowered the expression of these inflammatory cytokines and chemokines in chronic pancreatitis (*p < 0.05*).

The fold change of mRNA expression of TGF-β, PDGF-β and active hedgehog signaling Shh and GLI1 were also quantitated with a real-time PCR assay. As shown in Figure 4C-F, compared to the control mice, significantly increased Shh and GLI1mRNA expressions were observed in the pancreatic tissues of caerulein induced chronic pancreatitis mice (p < 0.05). More than 5 folds decrease of Shh and 2 fold decrease of GLI1 mRNA expression was found in the pancreatic tissues of sulindac treated chronic pancreatitis mice *(*p < 0.05). Expression of TGF-β and PDGF-β was also decreased in caerulein induced chronic pancreatitis mice fed with a diet containing sulindac.

To further confirm the qPCR results, the protein level of the above molecules were examed with western blot assay. Similar as the results of qPCR assay, as shown in Figure 4H, the protein expression of PDGF-β, TGF-β, SHH and GLi1 were all up regulated in caerulein induced chronic pancreatitis mice, significantly decreased expression of these proteins were observed in chronic pancreatitis mice with sulindac treatment (p < 0.05).

### Oral administration of sulindac inhibits PSCs activation as measured by α-SMA activity in chronic pancreatitis

To evaluate the activity of PSCs, the fold change of mRNA expression of α-SMA,was quantified with a quantitive real-time PCR assay. As shown in Figure 4G, compared to the control mice, the mRNA expression of α-SMA was up regulated in the pancreatic tissues of caerulein induced chronic pancreatitis in mice, sulindac treatment markedly decreased the expression of α-SMA at the mRNA level for about 2 folds. The protein level of α-SMA expression was also measured by western blot assay, and the result was accordant with our qPCR result (Figure 4H).

### Inhibition of MAPK signaling pathway in mice with chronic pancreatitis treated with sulindac

**Figure 5:**
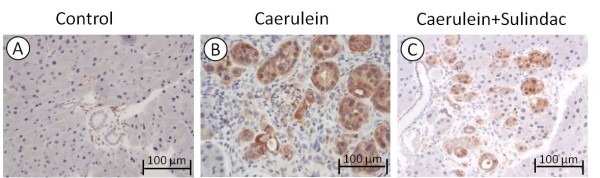
**Immunohistochemical analyses of phosophorylated ERK expression in chronic pancreatitis.** (**A**) No p-ERK expression in control mice; (**B**) High intensity of p-ERK expression in *caerulein induced chronic pancreatitis* mice; (**C**) Significantly decreased p-ERK expression in *caerulein induced chronic pancreatitis* mice with sulindac treatment.

Phosphorylation of ERK1/2 protein (p-ERK) as a key molecule in ERK cascade is well detected immunohistochemically. No staining signal was detected in the pancreas in control mice (Figure 5A). Acinar-ductal metaplasia lesions in chronic pancreatitits were strongly stained by the anti-p-ERK antibody in caerulein induced chronic pancreatitis mice (Figure 5B). A marked decrease of staining intensity was seen in chronic pancreatitis mice treated with sulindac (Figure 5C).

**Figure 6:**
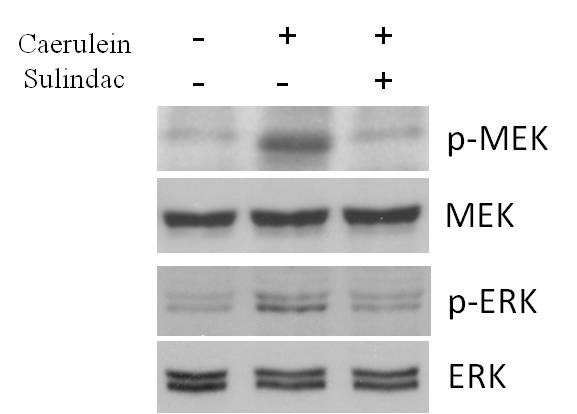
Western blot assay of MAPK signaling pathway in pancreatic tissues: Sulindac treatment exhibited a significant reduction of the phosphorylation of MEK1/2 and ERK1/2 proteins expression in the pancreatic tissues of caerulein induced chronic pancreatitis mice.

The activity of Phosphorylation of MEK1/2 and ERK1/2 was further analyzed quantitatively using western blot approach. As seen in Figure 6, using total MEK1/2 and ERK1/2 proteins as control, sulindac treatment exhibited a significant reduction of phosphorylation of MEK1/2 and ERK1/2 proteins comparing with caerulein induced chronic pancreatitis mice.

## Discussion

Chronic pancreatitis is resulted from chronic repetitive inflammation within the pancreas, resulting in recurrent repair of the pancreatic damage and ultimately in activation of a profibrotic cascade [32]. In the present study, we demonstrated that sulindac significantly inhibited chronic pancreatitis and pancreatic fibrosis in caerulein induced chronic pancreatitis mouse model.

Inflammatory cell infiltration (including neutrophiles, macrophages, lymphocytes and plasma cells etc.), a complex chronic active inflammatory process, is a character of chronic pancreatitis [31]. The inflammatory cells play a crucial role in causing tissue injury (acinar loss) and fibrosis either through active inflammatory cell directly induced tissue damage or via production of oxidative free radicals, inflammatory mediators or cytokines [26]. Our results showed that inhibition of chronic pancreatitis by sulindac was through a significant reduction of myeloperoxidase-labeled neutrophils and Mac-3-positive macrophages infiltration in the pancreas which is also paralleled with significant decrease of pancreatic injury as measured by amylase and cytokeratin-19 and Masson trichrome staining labeled pancreatic fibrosis. Our studies demonstrated a significant decrease of inflammatory cells including neutrophils and macrophages infiltration in the pancreas could be one of the key mechanisms for chemoprevention of pancreatic inflammation and fibrosis by sulindac.

TNF-α is the best characterized pro-inflammatory cytokine that is mainly secreted by macrophage [33-36], MCP-1 is a chemokine recruits monocytes, memory T cells, and dendritic cells to sites of tissue injury, infection, and inflammation [37]. These inflammatory mediators play important role in the course of inflammation. In our experiment, the expression of these inflammatory cytokines or mediators in the pancreas of chronic pancreatitis mice were quantified with q-PCR assay and showed that sulindac treatment significantly decreased the expression of TNF-α and MCP-1 mRNA in the pancreas. These findings imply sulindac treatment contributes to the attenuation of inflammatory cell infiltration by reducing these key inflammatory mediators and cytokines.

Fibrosis and desmoplasia are the characters of chronic pancreatitis and pancreatic stellate cells play a crucial role in pancreatic fibrogenesis in chronic pancreatitis [7]. Aoki et al. found that COX-2 is constitutively expressed in activated PSCs, and COX-2 inhibitors blocked the activation of quiescent PSCs and upregulation of α-SMA expression *in vitro*, indicating that COX-2 is important in the initiation and promotion of PSC activation [20]. The expression of α-SMA, which indicats the activation of PSCs was significantly upregulated in the chronic pancreatitis mice in our study, sulindac treatment resulted a marked reduction of α-SMA expression, implying that targeting the PSCs could be one of the key mechanisms for the anti-fibrogenic effect of sulindac.

TGF-β and PDGF-β, liberated by inflammatory cells, have been shown to have significant impact on the activity of PSCs [3]. In our experiment, the expression of profibrogenic inflammatory cytokines TGF-β and PDGF-β were upregulated in caerulein induced chronic pancreatitis, and a reduction of TGF-β and PDGF-β expression were found with sulindac treatment. Although the decrease of TGF-β and PDGF-β mRNA expression in caerulein induced chronic pancreatitis mice fed with sulindac diet did not reach statistical significance, the protein expression of TGF-β and PDGF-β were upregulated in chronic pancreatitis mice and sulindac treatment significantly decreased TGF-β and PDGF-β expression at the protein level. Therefore, the attenuation of profibrotic inflammatory cytokines TGF-β and PDGF-β could be a mechanism for the regulation of PSCs activation by sulindac.

The expression of Shh/GLI activity was also significantly increased in the pancreas of caerulein induced chronic pancreatitis mice, and sulindac treatment showed a markedly down regulation of Shh and GLI1 mRNA and protein expression, implying that targeting Shh/GLI pathway by sulindac could be a mechanism for inhibiting pancreatic fibrosis and desmoplasia in chronic pancreatitis. Our results imply that the aberrant Shh/GLI1 pathway could be involved in pancreatic desmoplasia in caerulein induced chronic pancreatitis.

A major pathway for regulation of the progression of chronic inflammation and fibrosis in chronic pancreatitis is extracellular signal related kinase (ERK1/2) pathway that is related to mitogen-activated protein kinase (MAPK) [38]. In this study, using immunohistochemical and Western blot approach, we found that sulindac treatment inhibit the phosphorylation of MEK and ERK, the key molecules in MAPK signaling pathway which plays a pivotal role in the activation of cellular processes such as proliferation, differentiation, and oncogenic transformation. This result indicated that the inhibition of chronic pancreatitis and consequent pancreatic carcinogenesis by sulindac maybe by targeting the ERK/MAPK signaling pathway.

## Conclusions

Taken together, we provide the evidence that sulindac exhibits strong activity against inflammation and fibrosis in the context of chronic pancreatitis in mice via blocking inflammatory cell infiltration and the activation of pancreatic satellet cells. EKR/MAPK signaling pathways may be influenced in reduced chronic inflammation and fibrosis. Sulindac is a very promising reagent for inhibiting chronic pancreatitis and will have a high potential to translate into clinical trial in future for the chemoprevention of chronic pancreatitis and its associated pancreatic carcinogenesis.

## Abbreviations

CP: chronic pancreatitis; ROS: reactive oxygen species; RONS: nitrogen species; AA: arachidonic Acid; NSAIDs: non-steroidal anti-inflammatory drugs; COX: cyclooxygenase; PG: prostaglandin; IACUC: Institutional Animal Care and Use Committee; IP: intraperitoneal; CPI: chronic pancreatitis index; MPO: myeloperoxidase; ERK: Extracellular signal-regulated kinases; DAB: 3, 3-diaminobenzidine; AEC: 3-amino-9-ethylcarbazole; TNF-α: tumor necrosis factor α; MCP-1: monocyte chemotactic protein 1; TGF-β: transforming growth factor β; PDGF-β: platelet derived growth factor β; Shh: sonic hedgehog homolog; GLI1: glioma-associated oncogene homolog 1; GAPDH: Glyceraldehyde-3-phosphate dehydrogenase; DEPC: diethylprocarbonate; PVDF: polyvinylidene fluoride; BSA: bovine serum albumin; TBST: Tris-buffered saline with 0.1% Tween-20; MEK: MAP kinase kinase; HRP: horseradish peroxidase; RT: room temperature; CK19: cytokeratin 19; HPF: high power field; MAPK: Mitogen-activated protein kinases.

## Competing interests

The authors declare that they have no competing interests.

## Authors’ contributions

HB and XGD designed, executed and coordinated of the studies. HB, XKC and LZ carried out the experiments, HB and XKC drafted the manuscript and XGD supervised the draft of the manuscript. All authors read and approved the final manuscript.

## Pre-publication history

The pre-publication history for this paper can be accessed here:

http://www.biomedcentral.com/1471-230X/12/115/prepub
